# *Arthrobacter* and related genera: An annotated selection of World Wide Web sites relevant to the topics in environmental microbiology

**DOI:** 10.1111/1751-7915.12339

**Published:** 2016-01-06

**Authors:** Lawrence P. Wackett

**Affiliations:** ^1^ Microbial Biochemistry and BiotechnologyDepartment of BiochemistryMolecular Biology and BiophysicsUniversity of MinnesotaSt PaulMN55108USA

## The genus *Arthrobacter*

### http://link.springer.com/referenceworkentry/10.1007%2F0-387-30743-5_36#page-1

*Arthrobacter* are very common in many soils and are readily isolated in laboratory media and so they have been isolated extensively from soils. There are many species known and this chapter offers a detailed description of the *Arthrobacter* genus.

## *Arthrobacter*: Wikipedia

### https://en.wikipedia.org/wiki/Arthrobacter

This Wikipedia entry is limited but contains some useful information about *Arthrobacter* in general.

## *Arthrobacter*: Microbe Wiki

### https://microbewiki.kenyon.edu/index.php/Arthrobacter

This entry describes *Arthrobacter* species with an emphasis on the ability of many of the strains to transform anthropogenic chemicals into less toxic products.

## *Arthrobacter*: Nomenclature

### http://www.bacterio.net/arthrobacter.html

There are very many *Arthrobacter* species listed on this taxonomic site. *Arthrobacter globiformis* is the type strain.

## *Arthrobacter* species can grow anaerobically on nitrate

### http://www.ncbi.nlm.nih.gov/pubmed/12829291

At one time, it was considered that *Arthrobacter* are obligate aerobes, but this study demonstrated that some *Arthrobacter* can grow on nitrate as the final electron acceptor under anaerobic conditions.

## Actinobacteriophage database: *Arthrobacter*

### http://phagesdb.org/hosts/genera/3/

This database contains information on phages of Actinobacteria. The specific page referenced contains lists of phages of *Arthrobacter*.

## *Arthrobacter arilaitensis*

### http://www.genoscope.cns.fr/spip/-Arthrobacter-arilaitensis,551-.html

*Arthrobacter* are very often associated with cheese ripening and the most common species isolated is *A. arilaitensis*.

## *Arthrobacter chlorophenolicus*: Genome portal

### http://genome.jgi.doe.gov/artch/artch.home.html

*Arthrobacter chlorophenolicus* metabolizes chlorophenols, including the highly toxic pentachlorophenol. This page is the entry for information on the genome of this organism.

## *Arthrobacter chlorophenolicus*: Thesis

### http://pub.epsilon.slu.se/1751/1/Unell_Maria_2008_26.pdf

This link is for a thesis on the biodegradative strain *Arthrobacter chlorophenolicus*.

## *Arthrobacter aurescens* TC1: Genome atlas

### http://bacmap.wishartlab.com/organisms/434

*Arthrobacter aurescens* TC1 was isolated from a soil site that had been heavily contaminated from a spill of the herbicide atrazine. The genome of the organism consists of a chromosome and two large plasmids.

## *Arthrobacter* review article

### http://www.scirp.org/journal/PaperDownload.aspx?paperID=49677

This recent review article on *Arthrobacter* species provides good background information on the genus.

## *Arthrobacter* biodegradation pathways

### http://eawag-bbd.ethz.ch/servlets/pageservlet?ptype=allmicros

This list in the Biocatalysis/Biodegradation Database contains 22 *Arthrobacter* species involved in biodegrading a wide variety of compounds, e.g. nicotine, organosilicon compounds, fluorene, and the herbicide atrazine.

## Arthrobacter: PloS/ONE

### http://www.plosone.org/browse/arthrobacter

This page contains links to research on, or mentioning, *Arthrobacter* species.

## *Rhodococcus*: Wikipedia

### https://en.wikipedia.org/wiki/Rhodococcus

*Rhodococcus* are common soil bacteria, are known for having extensive catabolic activities, and have large genomes relative to most prokaryotes.

## *Rhodococcus*: Microbe Wiki

### https://microbewiki.kenyon.edu/index.php/Rhodococcus

This Wiki page gives a good overview of the genus *Rhodococcus*.

## *Rhodococcus*: Nomenclature

### http://www.bacterio.net/rhodococcus.html

There are a large number of *Rhodococcus* strains highlighted here. The type strain is indicated as *Rhodococcus rhodochrous*.

## *Rhodococcus equi*: KEGG genome

### http://www.kegg.jp/kegg-bin/show_organism?org=req

*Rhodococcus equi* is one of the rare pathogens in the genus *Rhodococcus* and it can cause disease in horses and goats.

## *Rhodococcus* biodegradation pathways

### http://eawag-bbd.ethz.ch/servlets/pageservlet?ptype=allmicros

This list in the Biocatalysis/Biodegradation Database contains 30 *Rhodococcus* species involved in biodegrading a wide variety of compounds, e.g. styrene, acetylene, caffeine, and dimethylisophthalate.

## *Micrococcus*: Wikipedia

### https://en.wikipedia.org/wiki/Micrococcus

*Micrococcus* has been most recently isolated from human skin and food but it is also found in soil, as indicated in this Wikipedia entry.

## *Micrococcus*: Taxonomy

### http://www.bacterio.net/micrococcus.html

*Micrococcus* strains are numerous. The type strain is *Micrococcus luteus*.

## Taxonomic dissection of the genus *Micrococcus*

### http://www.ncbi.nlm.nih.gov/pubmed/7547287

This study suggested that the genus *Micrococcus* be subdivided into *Micrococcus, Kocuria, Nesterenkonia, Dermacoccus*, and *Kytococcus*.

## Micrococcaceae: NCBI taxonomy

### http://www.ncbi.nlm.nih.gov/Taxonomy/Browser/wwwtax.cgi?mode=Undef&id=1268&lvl=3&lin=f&keep=1&srchmode=1&unlock

This page has extensive lists of bacteria in the genera *Arthrobacter, Kocuria, Micrococcus, Nesterenkonia, Rothia*, and others.

## A *Nesterenkonia* polyextremophile

### http://genomea.asm.org/content/2/2/e00197-14.full

This is a genome announcement for an Antarctic isolate that was shown to be tolerant of low temperature, high salinity, and high alkalinity.

## *Kocuria*: Taxonomy

### http://www.bacterio.net/kocuria.html

*Kocuria* sp. are most like *Micrococcus* species and a number of the strains were previously considered *Micrococcus* before reclassification. This page contains an extensive list of *Kocuria* species.

## *Kocuria rhizophila* DC2201: Genome atlas

### http://bacmap.wishartlab.com/organisms/688

*Kocuria* are found in diverse environments. This isolate was found in a plant habitat and its genome provided new insights into this genus.

